# Refining Prescription Warning Labels Using Patient Feedback: A Qualitative Study

**DOI:** 10.1371/journal.pone.0156881

**Published:** 2016-06-03

**Authors:** Olayinka O. Shiyanbola, Paul D. Smith, Sonal Ghura Mansukhani, Yen-Ming Huang

**Affiliations:** 1 Division of Social and Administrative Sciences, School of Pharmacy, University of Wisconsin-Madison, Madison, WI 53705, United States of America; 2 Department of Family Medicine and Community Health, School of Medicine and Public Health, University of Wisconsin-Madison, Madison, WI 53705, United States of America; University of North Carolina at Chapel Hill, UNITED STATES

## Abstract

The complexity of written medication information hinders patients’ understanding and leads to patient misuse of prescribed medications. Incorporating patient feedback in designing prescription warning labels (PWLs) is crucial in enhancing patient comprehension of medication warning instructions. This qualitative study explored patient feedback on five newly designed PWLs. In-depth semi-structured face-to-face interviews were conducted with 21 patients, who were 18 years and older, spoke English, and took a prescription medication. These patients were shown different variations of the five most commonly used PWLs-Take with Food, Do not Drink Alcohol, Take with a Full glass of Water, Do not Chew or Break, and Protect from Sunlight. The 60-minute interviews explored feedback on patient comprehension of the PWL instructions and their suggestions for improving the clarity of the PWLs. At the end of the interview, patient self-reported socio-demographic information was collected with a 3-minute survey and a brief health literacy assessment was completed using the Newest Vital Sign. Twenty-one patients completed the interviews. Most patients were female (n = 15, 71.4%) with ages ranging from 23 to 66 years old (mean: 47.6 ± 13.3). The mean health literacy score was 2.4 on a scale of 0–6. Qualitative content analysis based on the text, pictures, and placement of the PWLs on the pill bottle showed preferences for including ‘WARNING’ on the PWL to create alertness, inclusion of a picture together with the text, yellow color highlighting behind the text, and placement of the PWL on the front of the pill bottle. Although patients had positive opinions of the redesigned PWLs, patients wanted further improvements to the content and design of the PWLs for enhanced clarity and understandability.

## Introduction

The Institute of Medicine estimates that at least 1.5 million preventable adverse drug events (ADEs) occur annually in the United States.[1] Complex and unclear written information on prescription labels have been found to be one of the leading causes of these preventable ADEs.[2–6] It is critical to examine the prescription labels that are placed on dispensing pill bottles because most patients rely heavily on the written information provided with their medicines. Without clear and simple instructions on how to take their medicines, these patients are subjected to medication errors that may lead to poor adherence and sub-therapeutic outcomes.[7–10] Complexity of written information hinders patients’ understanding of their medications which then leads to misuse of prescribed medications.[3–5] Although there is research on the medication information printed on drug containers or written information distributed in the pharmacies, few studies have examined the quality and the accessibility of medication warning information provided to consumers. The Institute of Medicine has called for concerted efforts on the evaluation of cautionary information placed on prescription bottles.[1] This call has led to research on redesigning medication labels to simplify the language, provide explicit texts, and have distinguishable fonts; core elements for an ideal label design.[4, 7, 11–14]

Prescription warning labels (PWLs) are small, colorful stickers adjacent to the prescription label on dispensing bottles that remind or highlight the most important instructions for patient’s safe and effective use of medications.[15–17] For example, PWLs contain warning statements about specified medications such as ‘Do not take with alcohol’ or ‘Take with food’. [15–17] Variations of existing PWLs have been shown to be a source of confusion for medication use as they provide complex, contradictory and excessive information. There are currently no published guidelines or federal standards regarding the content and presentation style of PWLs. [8, 16, 18] However, recommendations have been made for the improvement of PWLs, including the development of patient-centered PWLs which is necessary to improve medication safety and the involvement of patients in the refining of PWLs prior to the development of standardized PWL guidelines. [19]

Patients with lower health literacy are at greater risk for misinterpreting PWLs.[8] Redesigning and improving PWLs for clarity and better comprehension is therefore both a health literacy and patient safety issue.[9] Recent studies have shown the importance of developing patient-centered PWLs to ensure that medication information is properly conveyed and understood by patients across all literacy levels.[8, 18] Few studies have explored the development and implementation of patient-centered PWLs.[20, 21] Most of the research conducted has focused on assessing consumers’ ability to comprehend warning information and have led to suggested improvements such as simplifying the words and content, and the use of pictorial icons that may benefit and lead to increased understanding for patients with low health literacy.[8, 16, 18, 22, 23] In a previous pilot study, we developed five new PWLs that promoted patient understanding by increased font and label size, explicit graphics and text, and colored background.[15] Compared to previously designed patient-centered PWLs, the newly developed PWLs not only considered the complexity of the text, but also included additional elements for label designs, such as size, icons, content, placement on the bottle, and color.[15]

Following our pilot study, the objective of this current study was to explore patient feedback on the newly designed PWLs and develop recommendations for future more understandable PWLs.

## Materials and Methods

### Sampling, Recruitment and Design

We used convenience sampling to recruit patients from a primary care clinic in Madison, Wisconsin. The primary researcher and study team met with the clinic manager to work out a plan to inform patients about the study for recruitment. The recruitment process included: (1) The study coordinator attended a staff meeting to explain the study to the clinic staff prior to starting recruitment, (2) On the day of patient recruitment, the study coordinator was assigned to a group within the clinic that is staffed with the same clinic staff. The clinic staff placing patients in the rooms handed study flyers to every English-speaking adult who met the inclusion criteria; (3) The clinic staff asked if the patient would like to meet with the study coordinator to learn more about the study and then notified the study coordinator of interested potential participants; (4) The study coordinator met with potential participants in a private room at the clinic to explain the study, review the study summary, assess eligibility, and determine whether the patient could give verbal consent to participate. The recruitment took place on multiple days until no new information could be elicited from participants who completed the interviews. The Health Sciences Institutional Review Board of the University of Wisconsin-Madison approved the study. The use of verbal consent was approved by the review board because the research presented no more than minimal risk of harm to patients. Patients’ verbal consent was documented on the audio-recorder used during the interview.

### Data collection

**Fig 1:**
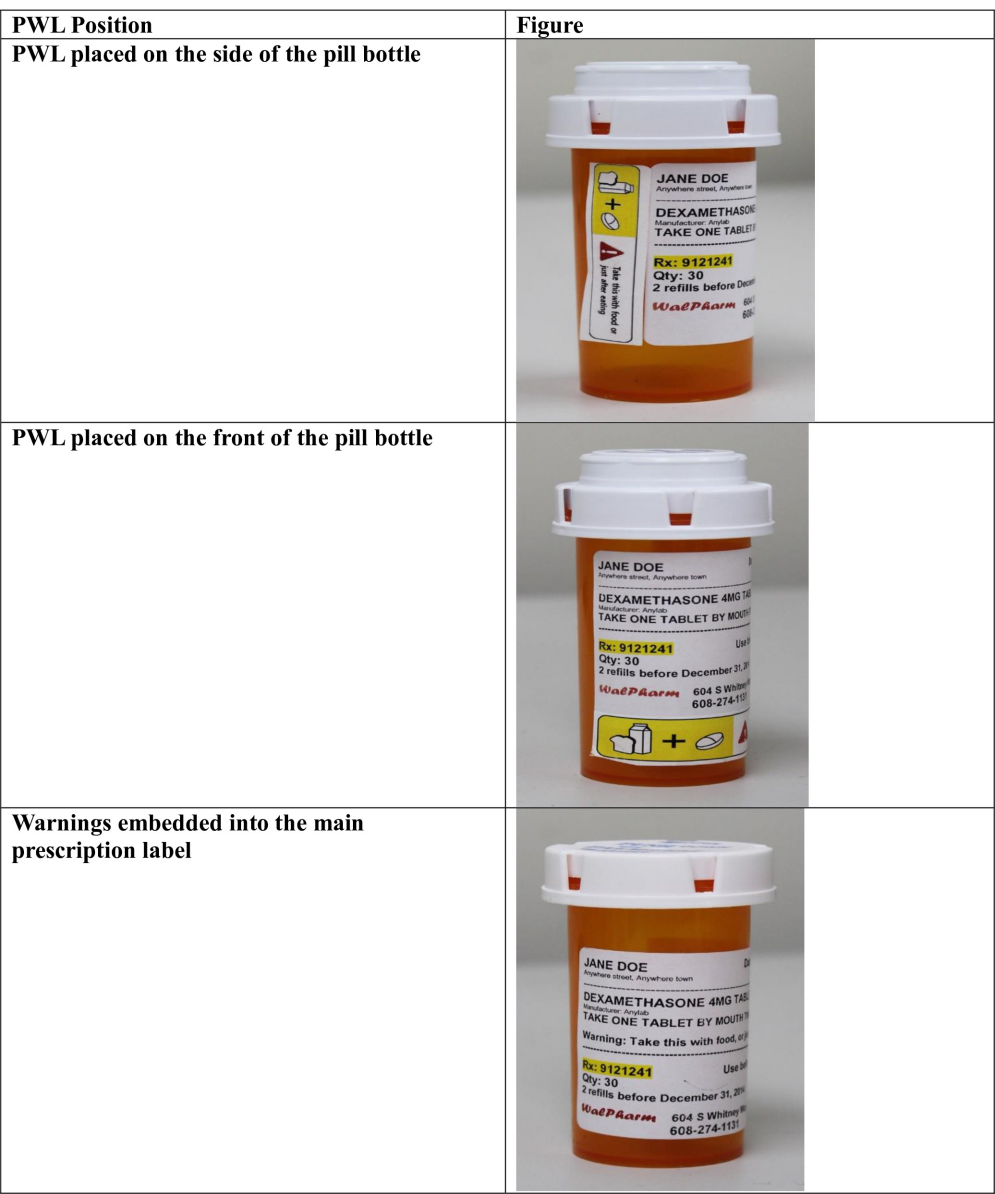
Positioning of the Prescription Warning Labels (PWLs) on the Pill bottle.

**Fig 2:**
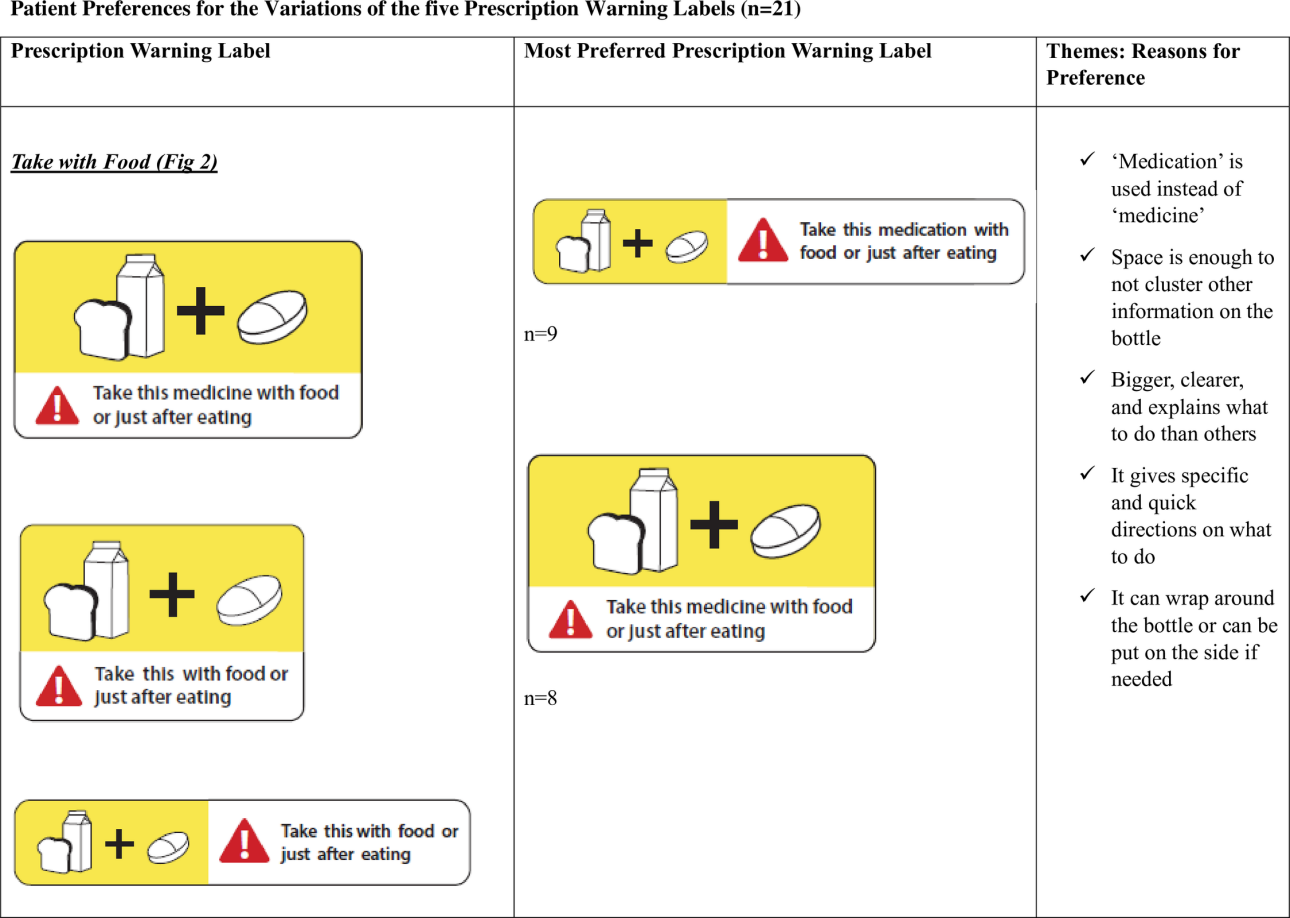
Take with Food-Patient Preferences for the Variations of the five Prescription Warning Labels.

**Fig 3:**
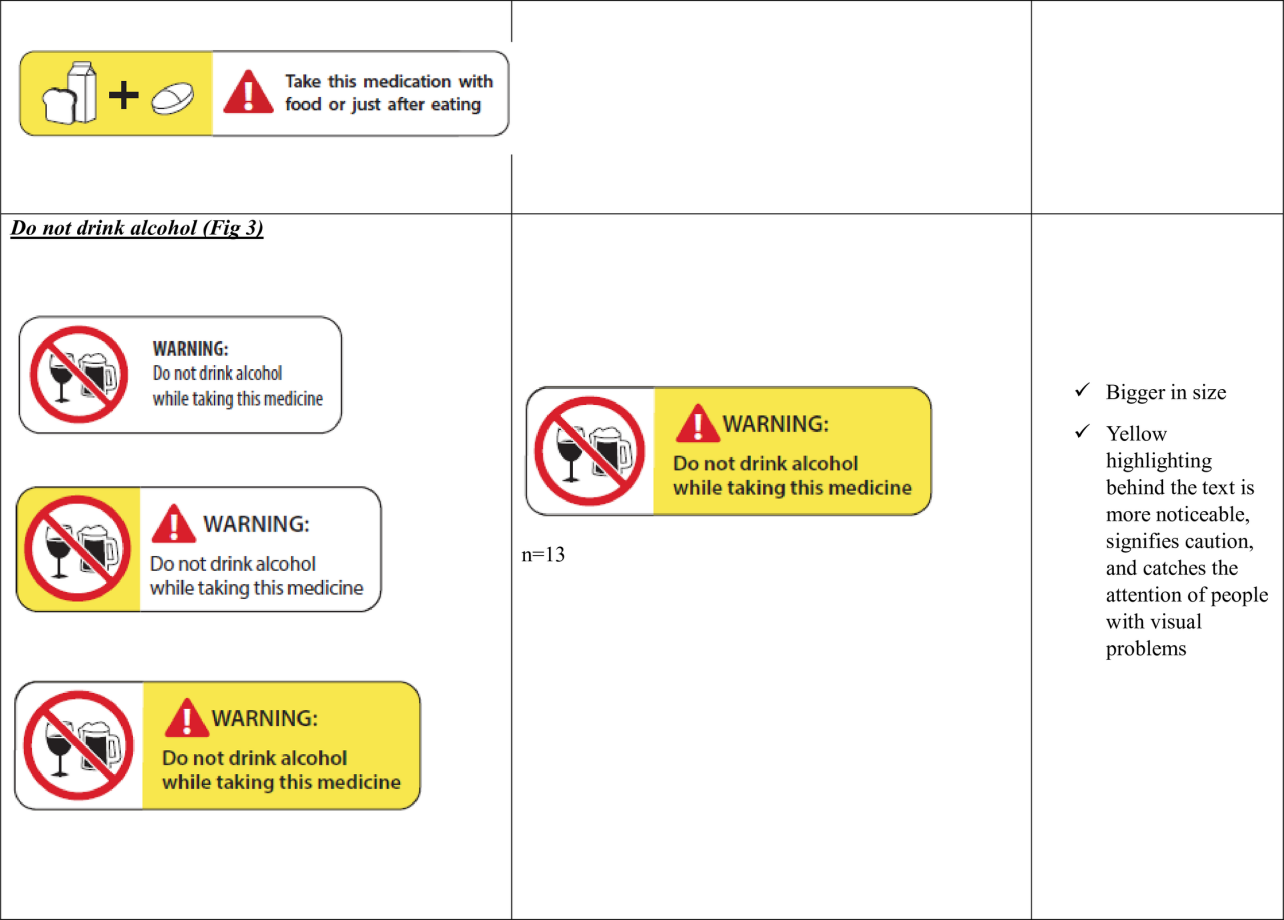
Do not drink Alcohol-Patient Preferences for the Variations of the five Prescription Warning Labels.

**Fig 4:**
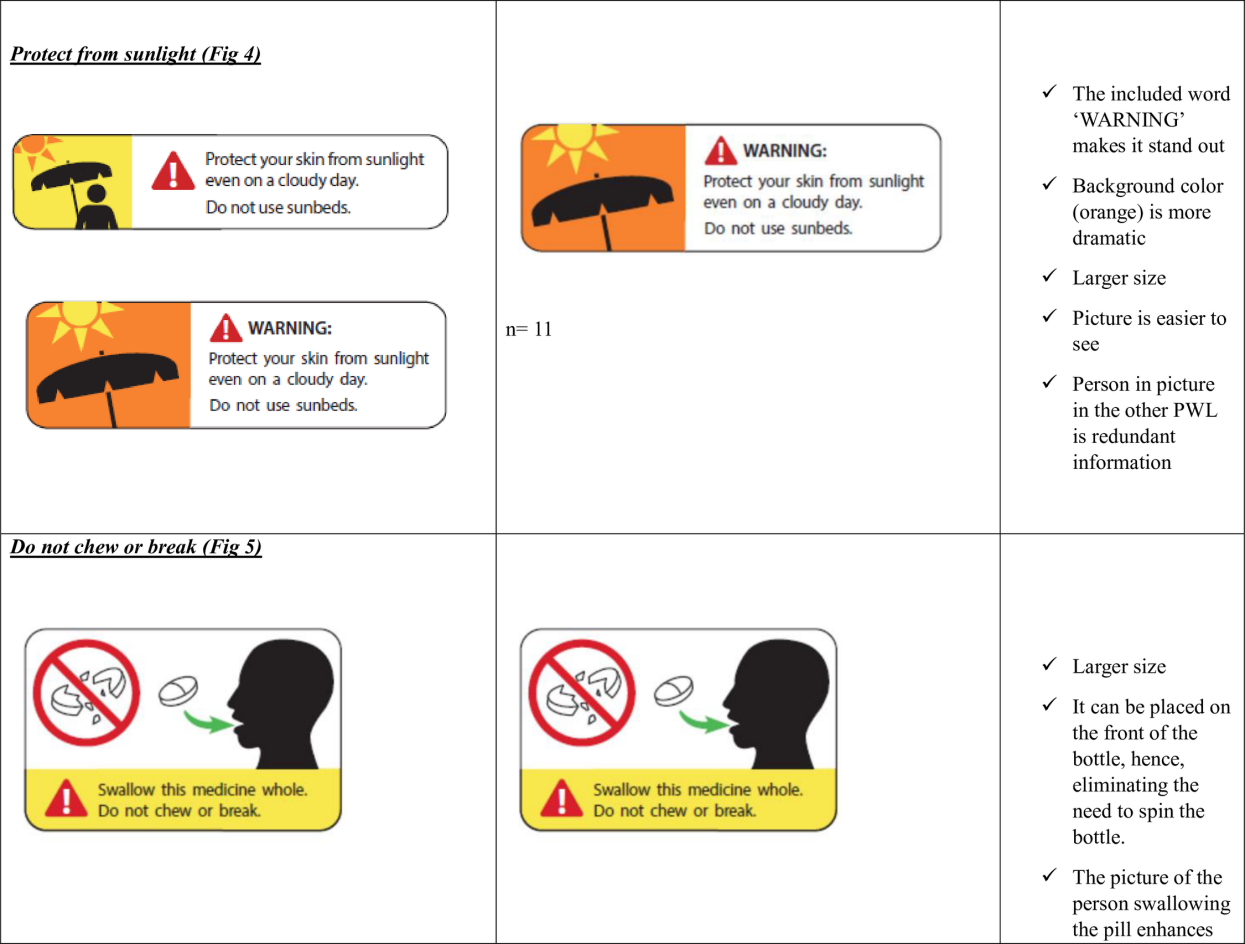
Protect from sunlight- Patient Preferences for the Variations of the five Prescription Warning Labels.

**Fig 5:**
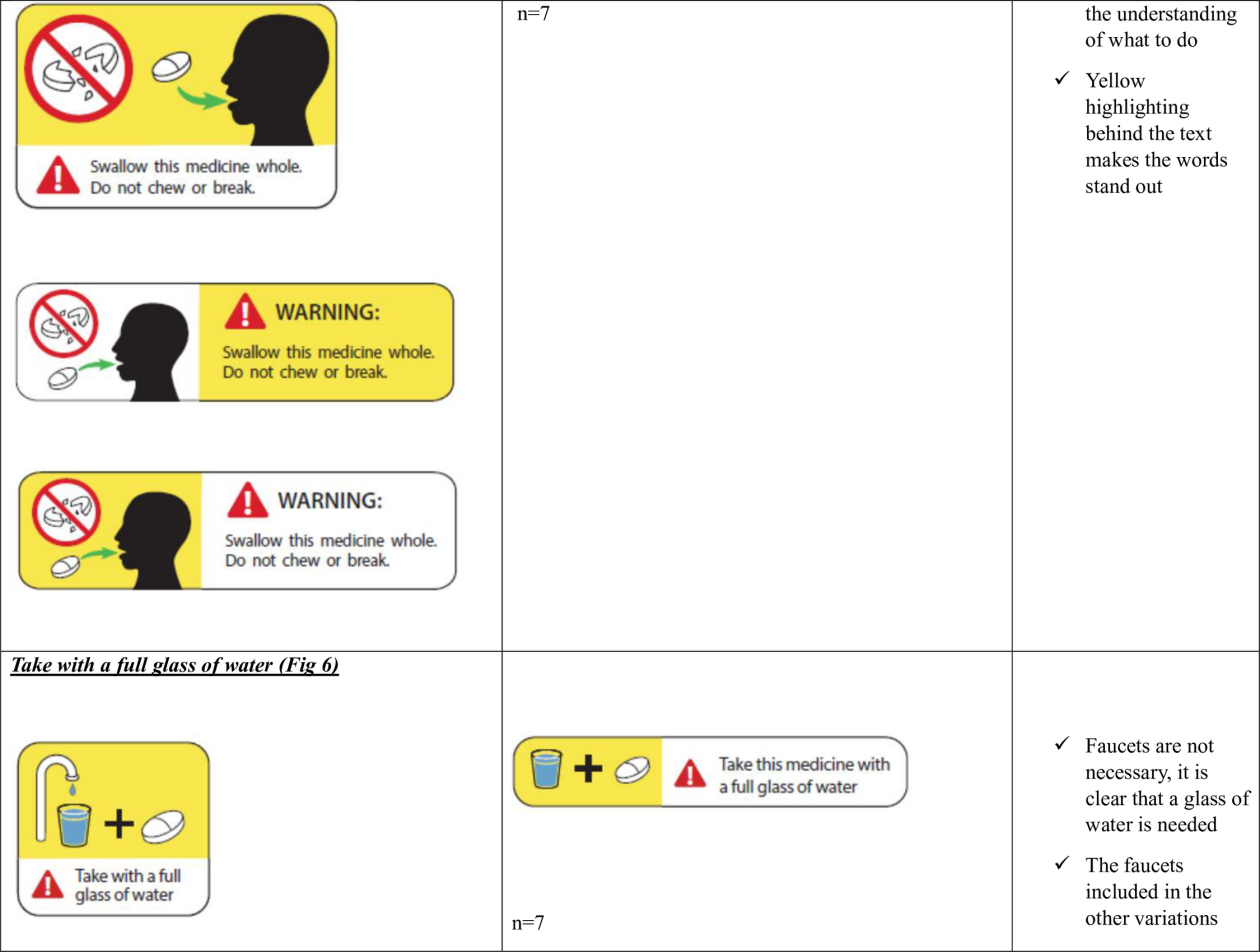
Do not chew or break- Patient Preferences for the Variations of the five Prescription Warning Labels.

**Fig 6:**
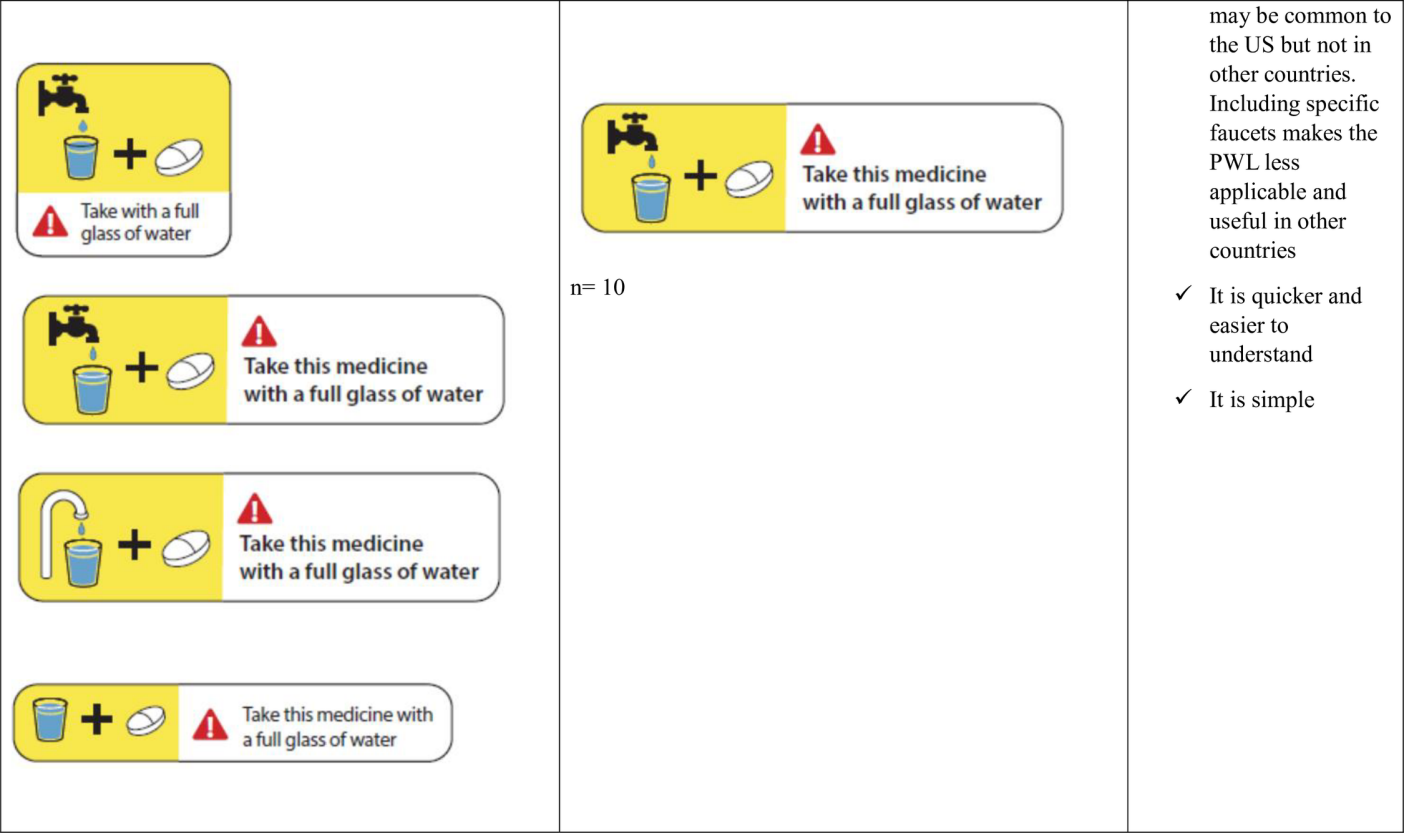
Take with a full glass of water- Patient Preferences for the Variations of the five Prescription Warning Labels.

Semi-structured 60-minute face-to-face interviews were conducted with adult participants who were 18 years and older, spoke English and took a prescription medication. This qualitative method was chosen because it allowed us to explore patient perspectives on the newly designed PWLs and gave the interviewer the flexibility to diverge from the questions when it was necessary to gain further patient insight. The interviews were completed between November 2014 and January 2015 and were facilitated by a trained female research specialist with a master’s degree in social work and five years of experience conducting qualitative research. The participants did not know the researcher prior to the study and the purpose of the research was communicated to them. Once the patient gave verbal consent, the interview took place on-site at the clinic with the primary researcher (OS) in the room sometimes for observations. The interviews explored patients’ interpretation, comprehension and feedback, including PWL positioning, (Fig 1) on the five newly designed PWLs. The PWLs included one of the following medication instructions:

1. Take with food (Fig 2)
2. Do not drink alcohol (Fig 3)
3. Protect from sunlight (Fig 4)
4. Do not chew or break (Fig 5)
5. Take with a full glass of water. (Fig 6)

During the interview, prescription bottles with a mock prescription label and a prototype of one PWL placed on the container were showed to the participant. Participants gave feedback on their comprehension of the PWLs content (text), pictures, and placement on the pill bottle. The interview questions were developed by two of the researchers (OS and PS) and piloted with five patients.

After commenting on one prescription bottle with a PWL label, patients were shown different variations of the five PWLs in a handbook with laminated sheets. For example, the PWL ‘Take with food’ had four variations with the same warning instruction worded differently. In some cases, a PWL variation had different pictures. Patients’ gave feedback on their interpretation of the text and pictures of each PWL and gave their preferences for each PWL variation. Questions also aimed to obtain additional information from patients regarding the graphics on the PWLs including their clarity and necessity. If the instruction on the PWLs was not clear or understandable, questions elicited what needed to be modified. Patients were also asked about desirable PWL placement and position on the bottle that would grab and hold their interest. The warning instructions were placed in three different positions on the pill bottles, (1) On a PWL on the side of the pill bottle, (2) On a PWL on the front of the pill bottle and (3) The warning instructions embedded into the main prescription label with no separate PWL sticker (Fig 1).

At the end of the interview, patient self-reported socio-demographic information was collected with a 3-minute survey and a brief health literacy assessment was completed using the Newest Vital Sign. [24] Participating patients received $50 as compensation for their time. All interviews were audio-recorded and transcribed verbatim by a certified transcriptionist.

### Data analysis

A qualitative content analysis of the open-ended interview responses identified common themes regarding patients’ interpretations, preferences for the PWL variations, feedback on each specific PWL, and preferred label placements. Using open coding, the analysis explored patient feedback across all five PWLs as well as specific feedback on each PWL. The analysis process included the following: initially reading the transcripts to achieve immersion, reading the data line by line to capture key thoughts, developing and organizing the themes and categories, and finally developing a conceptual model for how the themes were linked. [25]

Two project assistants (SM and YMH) coded the transcripts independently. A code book was not formally developed for the analysis. However, the two project assistants shared their initial codes, memos, and themes with each other. After several meetings with the coders and primary researcher, the final codes were chosen and listed in a word document. Similarities and divergences were discussed in meetings attended by both project assistants. Thereafter, the project assistants met with the researcher (OS) to review the themes. Agreement by consensus was reached on all themes before the results were interpreted. The transcripts were not returned to the participants for comments. NVivo 10 (QSR International-Melbourne) was used to organize and categorize the themes. Descriptive analysis was used to examine patient sociodemographic information. Chi-square tests were used to examine if patient PWL preferences differed by patient sociodemographic information or health literacy levels.

## Results

Twenty-one patients completed the interviews. Most patients were female (n = 15, 71.4%) with ages ranging from 23 to 66 years old (mean: 47.6 ± 13.3). The mean health literacy score was 2.4 on a scale of 0–6 with 4 patients (19%) with likely limited health literacy (score 0–1), 3 patients (14.3%) with possible limited health literacy (score 2–3) and 9 patients (42.9%) with adequate health literacy (score 4–6). The mean number of prescription medications taken daily was 6.6 (SD = 5.4) (Table 1).

**Table 1:** Descriptive statistics of the study population (n = 21).

Variable	Number (%)	Mean ± SD^a^
**Socio-demographics**		
***Age***		47.57 ± 13.26
***Gender***		
Male	6 (28.6)	
Female	15 (71.4)	
***Ethnicity***		
Hispanic	1 (4.8)	
Not Hispanic	20 (95.2)	
***Racial background***		
White Caucasian	8 (38.1)	
Black or African American	10 (47.6)	
Asian	1 (4.8)	
Mixed Race	1 (4.8)	
***Years of school completed***		
8 grades or less	0	
Some high school	4 (28.6)	
High school graduate or GED ^b^	6 (28.6)	
Some College	6 (28.6)	
College graduate	3 (14.3)	
Graduate degree	1 (4.8)	
***Health Insurance plan in the past six months***		
An individual plan	0	
A plan through your employer	5 (23.8)	
Military or VA^c^ Health Plan	1 (4.8)	
Medicaid	5 (23.8)	
Medicare	1 (4.8)	
More than one type of health insurance	6 (28.6)	
I have not had an insurance plan in the past 6 months.	2 (9.5)	
**Clinical characteristics**		
***Self-rated health***		
Excellent	4 (19.0)	
Very good	4 (19.0)	
Good	5 (23.8)	
Fair	5 (23.8)	
Poor	2 (14.3)	
***Number of prescription medications taken daily***		6.62 ± 5.39
1	2 (9.5)	
≥ 2	19 (90.5)	
***Number of pharmacies used in the past six months***		1.48 ± 0.75
1	13 (61.9)	
≥ 2	8 (38.1)	
***Health literacy level***		
High likelihood of limited health literacy	4 (19.0)	
Possibility of limited health literacy	3 (14.3)	
Adequate health literacy	9 (42.9)	

a. SD = Standard deviation units

b. GED = General Educational Development for Certificate of High School Equivalency

c. VA = Department of Veteran Affairs.

### Content and Pictures

Patient feedback on the content and pictures of the PWL are classified into three categories: (1) Preferences for the variations of the PWLs, (2) Feedback across all five PWLs (Table 2), and (3) Feedback on improvement of each specific PWLs (Table 3).

**Table 2:** Patient Feedback across all Five Prescription Warning Labels (n = 21).

Themes	Quotes
Bigger is better	“Like they, those are just, and they will probably have it on the side of a bottle, so you have to literally spin the bottle around to find the warning label. But the big one is just in a big spot, and you can see it much better. And you can read it much better.” (*Pt 12*)
Front placement is better because the importance of the warning is associated with its positioning on the label	“(By placing the PWL on the side)….Not that it's less urgent, it's just that it's, I don't think it's that. I mean, it's important. But it's not going to kill you if you don't. You'd probably have a stomachache. . .” *(Pt 8)*
Combination of instruction and picture is better	“I think to hit all, everybody, I think both because some people are stimulated by pictures, and some are by words. So I think you'd be able to hit both groups of people.” *(Pt 1)*
Yellow highlighting draws attention	“…this one, the letters, the instructions are not that big, and so they highlighted with the yellow. That’s perfect…You know, highlights that, what it's saying, what it means.” (*Pt 21*)
The word “WARNING” is alerting	“It says, warning, very clear if it says warning, that should get your attention right away. The one that says do not, that’s another thing that should get your attention, do not drink alcohol while taking this medicine. The warning is very clear in the sense it’s telling you, listen, this is something that’s like poison.” (*Pt 18*)
Make the picture more pronounced for clarity	“Maybe if the person was down a little more to the bottom you'd have more room at the top to put more of the sun up there so you could see very clearly that that's the sun.” (*Pt 5)*
Make the instruction and the picture fully match each other	“But then why are you showing milk? Because it's not a food. It's a drink, per se.” “but I'm like the wording actually doesn’t match what the symbol shows.” (*Pt 16*)
Include the reason for the warning	“well, what if I take it without the food is, you know, am I going to start getting violently ill” (*Pt 16*) “…to be more specific about what a medication does to you besides just saying that you can’t take, or you can take with water. Do not chew this or whatever. It needs to be, you know, extended, more information. (*Pt 11*)

Legend

Pt = Patient.

**Table 3:** Patient Feedback on the Improvement of Each Specific Prescription Warning Label.

Label Name	Content (Words)	Pictures	Placement
**Take with Food**	**Format**	**Format**	**Format**
	Big size	Big size	Front is better
	Big font	Yellow highlight	
		Bolder	
	**Content**	**Content**	**Content**
	Make food pronounced	Plus sign is confusing	
	Timeframe for taking the medicine is absent	Red exclamation point is unclear	
	Add “Warning”	Add “MILK” to the picture	
	Add “MILK” to the content	Replace with Bowl of fruit	
	Omit “just” and say after eating	Why milk, milk is not food?	
	Include reason behind the warning?	Timeframe for taking food is not shown	
		Picture depicts only breakfast	
**Do not chew or break**	**Format**	**Format**	**Format**
	Bigger size	Bigger size	Front is better
		Introduce color differences between the two pills	
	**Content**	**Content**	**Content**
	Include reason behind the warning	Add a drink to avoid confusions that water is not needed to swallow the medicine	
	Add “Talk to your doctor, if it happens”	Unclear description of the broken pill- “broken glass”, “food”	
	Add “do not crush”	Depict “don’t chew”	
	Include reason behind the warning	Remove the pill with a cross	
	Add “Talk to your doctor, if it happens”	Person not required	
	Add “do not crush”	Person should open their mouth	
		Add a cancel sign to capsule	
		Show a capsule too	
**Protect from sunlight**	**Format**	**Format**	**Format**
	Make tanning beds bold	Entire picture should be bigger	Front is better
	Make it bigger	Orange is more highlighting	
	Red warning is important		
	**Content**	**Content**	**Content**
	Change sunbeds to tanning beds	Make sun more prominent	
	Add an association between the medicine and warning	Umbrella associated more with rain than sunshine	
	Add sunscreen	Include circle with a sun and a cross though it	
	Include the reason for the warning	Picture looks like a beach	
	Add the word “WARNING	Add sunscreen or a person with a hat	
	Tanning bed is not associated with sun	Doesn’t need an umbrella	
	Add light from sun and UV rays	Delete waves in the background	
		Include circle with a cross on UV rays	
		Doesn’t depict cloudy days	
		Looks like a canopy	
**Do not drink Alcohol**	**Format**	**Format**	**Format**
	Make bold	Red triangle is unclear	Front is better
	Make bigger	The warning sign should be closer to the print	
	**Content**	**Content**	**Content**
	Include the reason for the warning	Add a cocktail glass	
	Unclear if alcohol can be taken later in the day	Make the bottle of alcohol full	
		Use STOP signs	
		The cross between the wine and the beer is confusing	
**Take with a full glass of water**	**Format**	**Format**	**Format**
	Big size	Big size	Front is better
	Linear order	Yellow highlight	
	Bold	Not on the side	
		Linear order	
		Bold	
	**Content**	**Content**	**Content**
	Define “full glass”	No faucet	
	Red triangle is serious	Bigger glass	
	Doesn’t seem important	Change faucet	
	Add “Warning”		
	Include reasons for the warning		

#### Preferences for the variations of the PWLs

Patients had preferences for the PWLs variation shown to them and there were specific rationale for their choices. The PWL preferences did not vary by participants’ sociodemographic information or health literacy level (results not reported).

The ‘Take with food’, ‘Take with a full glass of water’, and ‘Do not chew or break’ PWLs had the least clear patient preference. For the ‘Take with food’ label, nine patients chose the elongated label whereas eight patients chose the label with the larger surface area (Fig 2). Patients who chose the longer elongated label preferred this variation because of its ability to be wrapped around the pill bottle and cover less space on the bottle which seemed practical. Other patients chose the latter label because of its bigger size.

Patients preferred a specific variation of the ‘Do not drink alcohol’ PWL (Fig 3) and Protect from sunlight’ (Fig 4) and had distinct reasons for their preferences. Patient preferences for the ‘Do not chew or break’ PWL (Fig 5) varied widely but there were no distinct reasons for patient preferences.

For the ‘Take with a full glass of water’ label, patient preferences varied between the PWL with the picture of a faucet and the PWL without the picture (Fig 6). Patients thought the PWL with the faucet was quicker to understand while other patients thought the addition of a faucet was unnecessary and the specificity of the faucet would not allow the PWL to be used widely across countries.

#### Feedback across all five PWLs

Across all five PWLs, there were common major themes related to patient preferences.

Bigger size

Patients’ attention were drawn to labels which were bigger in size. Smaller elongated PWLs were linked to being placed on the side of the bottle versus the front of the bottle, which was not preferred by patients. (Table 2)

*“I would like bigger*, *bigger label*, *because they draw your attention more*. *The little ones*, *people can barely like read or see*. *I just feel like the bigger ones*, *you know*, *catches people attention*.*’* (Patient 2)

Including the word “WARNING” creates alertness

Patients expressed that the word ‘WARNING’ drew their attention to the PWL and it made them think of the cautionary instruction.

Combine both pictures and text in the PWL

Patients expressed that viewing the picture or text alone were not as effective as having them both together. Patients mentioned that including both the text and the picture would be helpful in targeting varied populations such as people with limited English proficiency and older adults with poorer eye sight. Also, participants thought that the combination of the text and the picture allowed for increased usability by all patients as some individuals seem to learn better with pictorial information while others may learn better with text (Table 2). The pictures also served as information back-up in case the content was confusing.

*“I feel more comfortable seeing the icon next to the words for the instruction*. *I like having the picture as backup*.*”* (Patient 19)

Including one main color (yellow) on the PWL

Patients thought the coloring of the text or pictures in yellow highlighted or emphasized information to them.

*“The picture is*, *you know*, *it has that yellow color that resembles*, *that actually attracts my vision more*.*”* (Patient 20)

Other minor themes included ensuring the content (text) matched the picture on the PWL, and including a reason for the warning instruction. For example, patients expressed that they wanted the label to state what would happen to them if they did not adhere to their medication warning instruction and took their medication with alcohol.

*“But*, *I mean*, *it doesn't really tell me anything specifically about the medication necessarily*. *It's just telling me*, *it's not a good idea to drink*. *So why or why not?”* (Patient 1)

Placement

Patients preferred the PWLs to be positioned on the front of the bottle. Patients thought the importance of the warning was associated with its positioning on the label.

*“Because when you*, *I mean*, *it's right there*. *Right in front of you when you see it*. *This one is noticeable*, *but*. *(others) you would have to turn it to the side*.*”* (Patient 3)

#### Feedback on the improvement of each specific PWL

Patients gave their feedback on one selected PWL variation. Their feedback specifically targeted the written instruction (content), the picture, and the placement of each PWL on the pill bottle. Patients’ feedback varied depending on the specific PWL being evaluated.

1. Take with Food: *“Take this medication with food or just after eating”*

Patients expressed that the format of the label needed to be bigger and bolder and the use of yellow highlighting needed to be more pronounced. Some patients thought the words were clear and did not require any significant changes. Other patients thought that the wording was unclear and needed to be modified to include some changes (Table 3). For example:

Patients expressed that the label needed to clarify how much time could be identified as “just after eating.”

*“…*, *as far as a span of time*, *you know*, *take this within a half hour after eating or something*. *That would be more clear for someone who might overthink things*.*”* (Patient 19)

Patients wanted the warning instruction to explicitly state milk on the label if the picture had the icon of a milk carton on it.

*“I don't know if there's any way of getting the word milk on this carton because we don't have really cartons of milk anymore*. *They're all plastic now*…*”* (Patient 1)

2. Do not Chew or Break: *“Swallow this medicine whole*. *Do not chew or Break”*

Patients thought that the label had clear written instructions, however some patients felt that the instruction could be clearer and less ambiguous. For example, to enhance the clarity of the instructions, patients thought “do not crush” needed to be added to the instructions.

*“And the warning label is just to let them know do not crush the pill*. *So maybe it could just leave that on there*…*”* (Patient 3)

Patients stated that the instruction of the picture was unclear and the broken pieces of the pills appeared to be a broken glass. (Table 3). There were mixed responses on whether the graphic depicting a person in the PWL was needed.

*“you don't really need that*, *the head there”* (Patient 10)

3. Protect from Sunlight: *“Protect your skin from sunlight even on a cloudy day*. *Do not use sunbeds”*

Patients thought that this PWL was the least clear of all the PWLs. For example, most of the patients could not comprehend the word ‘sunbed’ and wanted it changed to ‘tanning beds’.

*“I never heard of that*. *Usually*…*they say sunscreen*. *But what is sun bed?”* (Patient 10)

Most patients expressed that the umbrella would remind them of the rain rather than sunshine. Therefore, the pictorial icon needed to clearly indicate avoid sunlight.

*“Well*, *you know*, *I can see now this red part at the top is the sun*, *but with the umbrella*, *somebody might think it's rain because you got umbrella*. *And people will associate umbrella with rain more than sunshine”* (Patient 5)

4. Do not Drink Alcohol: *“Do not drink alcohol while taking this medicine”*

Patients understood the seriousness of the PWL, but wanted more clarity on whether the medication should not just be taken with alcohol at that specific time of medication administration and if alcohol could be consumed after a certain period of time.

*“ah*…*but you might be able to drink alcohol later on in the day and you might still be able to drink*, *so”* (Patient 5)

Overall, the picture was well understood by patients; however, some patients wanted a martini glass added to make the picture even clearer.

*“I'd put a martini cup on there too*.*”* (Patient 7)

5. Take with a Full Glass of Water: *“Take this medicine with a full glass of water”*

Patients wanted the full meaning of a full glass of water explained in more detail. There were also mixed perceptions on whether a faucet needed to be added to the picture on the label.

### Placement

There were no variations in preferences for the placement of the specific PWL on the bottle. The feedback was similar across all the labels. Patients wanted the PWL placed at the front of the pill bottle.

## Discussion

There was a wide variation of patient feedback regarding their PWL preferences and improvements for the PWLs. Overall, suggested improvements were focused on the rewording of content and design changes including color, size, clear pictures, removal of pictorial icons, etc. Patients also wanted the PWLs to be placed on the front of the pill bottle.

PWLs are currently not standardized. Hence, patient misunderstanding of medication cautionary information cannot be completely prevented until the United States Food and Drug Administration calls for the standardization of all PWLs, including a recommendation for the utilization of patient-centered PWLs. Meanwhile, in preparation for the implementation of this recommendation and proposed requirement for patient-centered PWLs, it is important to involve patients in the refining of PWLs. [19] Wolf et al., 2006 provided some directions for the development of new PWLs. It was recommended that patients be actively involved so that the icon design, content and formats are usable by all individuals. [19] As is evident in the breadth and depth of patient’s suggested improvements, involving patients was paramount to the refinement of the newly designed PWLs.

Consistent with previous studies, patients preferred PWLs with increased label size and a bigger font size. [21, 26–28] Also feedback related to design such as the use of color highlighting behind the words versus color highlighting behind the pictures show that it is important to also integrate information design while refining PWLs. [11] In the past, there has been a lot of focus on enhancing the comprehension of the written content of PWLs, and using pictures/icons to depict the instructions. Our study results show that utilizing color and embedding the color in the right way are other PWL details that are important to patients.

Using the right pictorial icons seemed important to patients. If pictorial icons do not match the written instructions, patients may get even more confused and have less understanding of how to use their medication. In addition, patients suggested the removal of certain pictorial icons as it led to more confusions. This feedback shows that just combining pictures and content in a PWL is not enough. Pragmatic picture-content messages that complement each other, can improve visual attractiveness, and is not confusing to patients are needed in the redesign of PWLs.[11]

Interestingly, patients wanted the word ‘WARNING’ included on the PWL to create more attentiveness to the cautionary information. Previous studies show that patients do not pay attention to the warning instructions on their PWLs. [7, 19, 29, 30] Based on the fear appeal theory, [31] patients’ may need more persuasive messages that arouse fear and directs their attention to the threat or harm associated with ignoring the warning instructions. A fear-based warning instruction may allow patients to feel vulnerable to the risk of a medication error or adverse drug event occurrence, allowing them to take protective actions such as paying attention to the instructions on their PWLs.

Patients wanted the reason for the warning information included on the PWL. This feedback makes two issues clear in the refinement process of PWLs. First, there is potential for a disconnect between patient preferences and the actual integration of feedback into the redesign of PWLs. [32] Given the limited size of most pill bottles, including the reason for the warning, while preferred by patients, may not be feasible. Second, it is possible that patients need more clarifications during the communication of written information. Simply placing PWLs on the pill bottle without additional justification of the need for the information may be critical to patients’ adherence to the instruction. Pharmacists might need to engage patients in more conversations about the medication warning instructions during counseling. In addition, websites, posters in pharmacies and other media could be used to communicate the importance of adhering to prescription warning information to patients.

Overall, patients wanted the PWL placed on the front of the pill bottle. Limited studies have focused on the positioning of the PWL on the dispensing bottle or ensuring that PWLs are visible to patients. In a previous study, it was noted that the starting point for creating an effective PWL should be to design a label whose placement and label characteristics are likely to attract patients’ attention. [22] It was suggested that only after such a label is developed can its impact be refined by subtle changes to wording. Sundar et al., 2012 further suggested that a focus for PWL redesign should be to create PWLs that attracts attention. The placement of the PWL on the dispensing bottle is vital to guiding patients’ attention. [22] Lee et al., 2013 showed that the placement of PWLs significantly impacts the probability that patients view warnings (p = 0.0011) and the amount of time that patients spend viewing information (p<0.0001). [33] Similar to our study that showed patient preference for the PWL placed on the front of the bottle, Lee and colleagues during their comparison between an interactive placement design (PWL on the top of the dispensing bottle cap), a horizontal placement (PWL at the front of the bottle), and a vertical placement (PWL on the side of the bottle) observed a higher probability of the PWL being noticed by either the interactive and horizontal placement. [33]

The identified improvements suggested by the patients in this study may have the ability to increase the quality and usability of the PWL by the patient, possibly leading to increased attentiveness to the PWLs during medication administration. However, it is important to acknowledge that patients’ suggested improvements to improve a PWL might not correlate with the ability to utilize all suggestions. For example, patients indicated that they wanted the pictures and content of the PWL to be bigger. Patients also wanted a sunscreen and a person with a hat added to the ‘Protect from sunlight’ label. While these suggestions are important, because of design limitations, actual incorporation into the PWL may not be feasible.

Some of the strengths of this study are that we had several participants with possible limited health literacy. Also, we had a high consensus of opinions on several PWL preferences. This study also had some limitations. The study participants were only English speaking and the revised labels were only written in English language. Future research should consider exploring the preferences of non-English-speaking patient populations. Twenty-one participants with a wide age range (23 to 66 years) precluded subgroup analysis by age. Patients in different age groups might interpret PWL pictures and content differently. Also, we did not explore specific feedback from individuals with colorblindness. The PWLs were not examined with patients using their actual medication containers. Patient feedback might differ if the PWL was on their own pill bottle. Our population was mostly women from one urban clinic in Wisconsin which limits generalizability. Future research should consider applying the preferences found in this study to revised PWL designs and test them in a broader audience and multiple sites across the country. An examination of these refined PWLs on patients’ medication use outcomes including adherence and recall of warning instructions is also needed. Patient feedback was only received regarding five PWLs. These five PWLs are the most common and frequently used PWLs in prescription drug labeling. Future research should consider the refinement of more PWL types and variations. Finally, we did not analyze the data to determine if patient understanding and interpretation of the PWL was correct or incorrect. Cognitive interviews with the patients were only used to pilot the interview questions but not to obtain patient feedback on the PWL. The results from this study could inform future research that could lead to prescription warning labeling policies and guidelines and help inform research that will lead to the standardization of PWLs. The study is important in further enhancing patient understanding of medication cautionary information, preventing medication errors and increasing patient safety.

## Conclusions

Patients had clear preferences for some of the newly designed PWLs but not for other PWLs. Patients preferred bigger and bolder content, color highlighting behind the warning instructions, and placement of the PWL on the front of the pill bottle. Although patients had positive opinions of the redesigned PWLs, they thought that further improvements to the content and design of the PWLs were required for enhanced clarity and understandability.

## Supporting Information

S1 Table Take with Food Label. (PDF)

S2 Table Do not drink Alcohol Label. (PDF)

S3 Table Protect from sunlight Label. (PDF)

S4 Table Do not chew or break Label. (PDF)

S5 Table Take with a full glass of water Label. (PDF)
