# Supplementary material for: Refining Prescription Warning Labels Using Patient Feedback: A Qualitative Study
Source: PLoS One. 2016 Jun 3;11(6):e0156881. doi: 10.1371/journal.pone.0156881 (PMC4892508; doi:10.1371/journal.pone.0156881)
Supplement: S1 Table — (PDF) [file pone.0156881.s001.pdf]

Fig 2: TAKE WITH FOOD LABEL:

Choices and Quotes	Memo
Label 4	
Pt 1	Big enough, Linear form of reading, space is enough to not bother other information on the bottle.
Pt 3: “it said take this medication and not just take this medicine. I think that's to me sounds better.”	Emphasis on specificity of instruction
Pt 4: “Because it says exactly what you are doing.” “Just not explaining what you are doing, what you are taking”. “What the difference is, this(1) is more smaller and this (4) is more bigger, and it's more clearer.”	The others aren’t explaining it well. more smaller more bigger, and it's more clearer.”
Pt 5: “This says specifically take the medication with food.”- “Its compact”	The words are much more actively speaking to the patients about their medication. The other ones are too big
Pt 8: “You know, and this seems to be more cut and dry.”<br>“Yeah. I think that's probably why because I'm used to seeing the longer ones for warnings. So maybe that's why my eye goes to that.”	Longer labels are assumed to be going on the side. Some sense of association between the longer labels and them being pasted on the side.
Pt 9: “It just comes through the quickest on what to do.”	
Pt 18: “it’s got the order, but it says that . . . really recognize, food plus medication, and then it says take this medicine with, like the rest of them, with food, or just after eating. It’s explanatory. It says tell, and then it says take the medicine with food, or just after eating. So it’s telling you a bunch of stuff. It’s got a picture. If you can’t read it, then you can look at the picture.”	Medication word is good, just after eating is good, picture is also good.<br><br>Likes wrap around labels more
Pt 19: “Because there’s no wasted space. Number three, there’s, you know, extra space. They could have notched the font up to push the word eating over more. Also, I respect the punctuation in number four. It’s the only one with a period, and I thought the punctuation was missing and have thought that throughout this whole group.”	Likes the punctuation on 4, Likes the picture and the wording combination , but feels that the picture is not that important in this context.
Label 1	
Pt 2: “If you see the picture, you get a better view of it and see what you need to take it with, food or water.” “Like they, those are just, and they will probably have it on the side of a bottle, so you have to literally spin the bottle around to find the warning label. But the big one is just in a big spot, and you can see it much better. And you can read it much better.”	Bigger, picture is clearly seen

Pt 6: "just take this medicine with food or just after eating. I mean, this sounds much better. The other ones is just, they're not good."	Likes the wordings
Pt 10: "Because it's bigger, and I can see it better"	The others are too long
Pt 12: "Because it's more like bigger, like people can see the small printing and, you know, the bigger one . . . especially if you got glaucoma, being sugar diabetic or so. But, me, I can see in the small thing, but I would prefer the bigger one for most sicker people or so you know"	Content was unclear because of the milk and the yogurt based products.
Pt 14: "It's bigger. It's direct and right to the point. It's not like you have to eat, you know, that you should eat before taking it instead of after you eat. So I think that's better.	The option of eating right after food is preferred
Pt 15: "Big, and, you know, you can read everything and see everything, which these get smaller. Some people with their glasses or no glasses, they might make a mistake, you know. Well, maybe not, but that's the one I would pick, yeah."	Number 3 is very small. Picked because it is bigger
Pt 20: "Just because it's a little bigger than the other ones. It has the words under the picture, so I can actually see both at the same time."	Explicitly "with the medicine",
Pt 21: "Because it's bigger, mm-hmm, and it's in there all together in one, you don't to . . . "	Bigger is better, doesn't need to turn to see, the picture is bigger too.
Label 3	
Pt 7: "smaller and more concise" "Because it doesn't say take this medication. But, I mean, that's common sense. You're taking a pill, you know, and then you have the picture. So, yeah."<br>"1 for people with sight problems"	Common sense that the label is talking about the medicine only.
Pt 16: "it's bigger, the bigger yellow like drew me right in. Because it was like, oh, there's something that I really need to know about this. The warning is bigger font as well. I mean, it's a little bit bigger sticker as well, but I think the warning is bigger. It brought my attention right to it immediately."	Yellow is attractive, more attention seeking
Label 2	
Pt 12: "it's, I guess the word's symmetrical. It's, it kind of stand out to me better. These are, the other three don't say read me, but this, number two says look at me to me."<br><br>"(3,4) yeah, you're going to turn it. You have to spin it all the way around to read it. That's probably your best one."	Symmetrical<br><br>Will have to turn the bottle for the longer labels, which is not preferred.
Pt 17: "Something about the format of having the picture streamlined with the written warning"	Number 2 is large enough. They are all easy to understand.

