# Supplementary material for: Refining Prescription Warning Labels Using Patient Feedback: A Qualitative Study
Source: PLoS One. 2016 Jun 3;11(6):e0156881. doi: 10.1371/journal.pone.0156881 (PMC4892508; doi:10.1371/journal.pone.0156881)
Supplement: S2 Table — (PDF) [file pone.0156881.s002.pdf]

Fig 3: Do not drink alcohol Label

Choice and Quotes	Memo
Label 1	
Pt 6: “Okay. So you like it without that.<br>RESPONDENT: Yeah.<br>INTERVIEWER: The triangle, the red triangle.<br>RESPONDENT: Yeah. I mean, it's telling you no right there, all of them. But it's just got the extra right there.<br>INTERVIEWER: Yeah. They all have the same picture.<br>RESPONDENT: Mm-hmm.<br>INTERVIEWER: Okay. So you don't think you need the extra colors and triangles?	Doesn’t like the red triangle and doesn’t think that the warning needs more exclamations or pictures
Label 2	
Pt 1: “And I like it because behind the alcohol, there's like an additional color and kind of another warning on top of the word warning and the red on the side.”<br>“So there's like more than one way to tell somebody that it's a warning.” “Well, I think both coming together I think is a good reinforcer.”	Appropriately sized, yellow behind the graphic<br>The yellow behind the graphic is highlighting it and the word warning and the exclamation point is highlighting the text.
Pt 9: “That's simplest”	Simple, appropriately sized.
Pt 12: “Because it's more clearer instead with the yellow printing or so. Most people can't see . . . color white or so, so the white printing would show more better than the yellow part.”	Yellow is better to have it behind the graphic as the text reads better with the white background.
Pt 13: “Number two's just right, not too big and not too small. And number one you said didn't stand out enough.”<br>“ if they're any bigger than this, then I'm thinking that they're going to be sideways, and then there's like seven of them like don't share your prescriptions, don't leave them so your dog's going to, you know, there's like all the silly ones, and then there's the serious ones, I guess.”	Size is appropriate<br><br>Larger size is associated with being put in the sideways direction and hence will not be useful. Especially because there are a lot of warning labels which can be silly. The important ones need to stand out in the front.
Pt 20: “The picture is, you know, it has that yellow color that resembles, that actually attracts my vision more.”<br>“Yeah, because in this case, it would still, you know, attract my vision because I know there is a red triangle there. But I like that the highlighting was to the picture and not to the wording.”	Yellow behind the picture is more attractive.<br><br>The red triangle is going to catch the eye anyway, so it reinforces the warning.
Label 3	
Pt 2: “: So once you see that is in yellow, bold writing, not really bold writing, but it's in yellow, that shows I shouldn't take it with like alcohol. It	Prefers big labels, yellow behind the text is preferred. States that the yellow behind the text

should click in your head not to. These people probably just don't pay attention to."	will catch the attention of people with visual problems.
Pt 3: Quote??	Yellow behind the text is more alerting
Pt 4: "And this one has that yellow color in it. I think you see this more."	
Pt 5: "It's got more yellow in it, and the size is okay. I like the fact that this is highlighting, the writing is highlighted."<br>"That could be, looks like a root beer. It remind me of root beer . . . this glass with a bottle, a real bottle, alcoholic bottle, I think that works better."	More the yellow, better would be the alertness. The red triangle and the warning sign should be separated. Likes highlighting if words<br><br>Getting rid of that mug, or, and replace it with an alcohol bottle.
Pt 7: " you know, it doesn't dummy itself down" "The triangle with the, you know, that says look at me" "Yeah. This is a real clear message."	The wording is strong, says it multiple times???<br>Triangle is important
Pt 8- "It's noticeable, more noticeable than white, the white"	Easier to see than a white background. Yellow under the text and the red alert warning
Pt 10: " Because it's the yellow, the warning sign."	Prefers for the warning sign to be in yellow. Yellow makes it stand out from other labels
Pt 14:"It's colorful, bright, bold, and to the point"	Yellow makes the label look important.
Pt 15: "It looked more of a warning type, you know." "Maybe it'll make them read it more with the yellow."	The idea of more yellow is better.
Pt 16: "I think, because it's bigger, the bigger yellow like drew me right in. Because it was like, oh, there's something that I really need to know about this. The warning is bigger font as well. I mean, it's a little bit bigger sticker as well, but I think the warning is bigger. It brought my attention right to it immediately."	The idea of more yellow is better, bigger font
Pt 17: " the red triangle with the exclamation point warning sign, you know, the warning sign, do not drink alcohol while taking this medicine. And you've got the picture right beside it. I like that one."	
Pt 18: " the red triangle with the exclamation point warning sign, you know, the warning sign, do not drink alcohol while taking this medicine. And you've got the picture right beside it. I like that one."	Yellow behind the words is sticking out more.
Pt 19:" I think that there's, simply that there is more yellow. Again, if this were in the number one size icon but the wording on the right was all filled with yellow, you'd still have more yellow than the other icon on number two. So I do think it's the fact that the wording is all surrounded by yellow. Yellow is caution. I think that the cancel sign on the alcoholic beverage is clear."	"I feel like punctuation is definitely deserved" its more respectful. ????

Pt 21: "Colorful, since it is colorful, you can, you know, your attention, like your attention is there. Oh, yeah, it's like, oh, you are, the message is there already, the attention, you know what I mean? "	Attention seeking??? because of the color

DONOT DRINK ALCOHOL LABELS
