# Supplementary material for: Refining Prescription Warning Labels Using Patient Feedback: A Qualitative Study
Source: PLoS One. 2016 Jun 3;11(6):e0156881. doi: 10.1371/journal.pone.0156881 (PMC4892508; doi:10.1371/journal.pone.0156881)
Supplement: S3 Table — (PDF) [file pone.0156881.s003.pdf]

Fig 4: PROTECT FROM SUNLIGHT LABELS

Choice and Quotes	Memo
Label 1	
Pt 1: “More linear looking”. “And it goes more with the sun versus I think of a sun as yellow. I don't think of a sun as orange.”	Number 2 is too big. Yellow color is more dependable for the sun than the orange
Pt 3: “Label 2 looked like Halloween decoration”	The colors on label 2 are not right
Pt 6: “Because it says, protect your skin from sunlight even on a dull day. Do not use sun . . . this one has got a warning.”	Clarity. Pays more attention to the words
Pt 9: “Quickest and easiest”	Quick gaze will inform what to do. Label 2 looks like a lot of reading.
Pt 12: “ , I got to take the pill to keep my body moist or so . . . and if people can't see that then they got problem when they taking their pills or so, you know.”	Connection of the high sun with drying??<br>Likes the picture of the person. The warning is clearer
Pt 18” I know the sun is shining, but this is telling the same sun is shining here and tells you the same thing, but I like the yellow”	Prefers the sun in the yellow. Thinks the information is the same in both.
Pt 19 “ imply based on the fact that it has yellow, not exactly for the purpose of it being a cautionary color but because the orange background blends in with orange bottles for number two, and I don’t think it would stick out as much.”	Orange blends in with the orange bottles, so yellow is preferred.
Pt 20 “ You can still see the sun, but it’s not a sunny, you know, it’s not bright.”	Orange is more highlighting, would prefer if the orange was on label 2 for background. Written word- warning in label 1???
Pt 21: “Number one is good because there is a person there, you. I’ve never seen this before. This is my first time to see a warning like this. Protect yourself to sunlight. You can do protect your skin from sunlight, or maybe you can just do like a lotion there, SPF 1, a picture of SPF 50, something like that. “	Likes the person figure<br>Suggestion is to ass sunscreen of SPF factors in there, add UV rays there
Label 2	
Pt 4:	The word warning
Pt 5: “more dramatic with the background color here” “ That was basically the color I first look at it, it has kind of an ominous feeling about it with that color scheme.”	Color scheme is attractive and yellow is a happy color. Orange is not
Pt 7: “more color difference”??	Larger size, most people use yellow for the sun
Pt 8 “brighter”	Larger size , brighter color scheme
Pt 10	Larger and orange is more warning like
Pt 11 “easier to see”	Picture on label 2 is easier to see, this pt has vision impairment

Pt-13 " I think the, like colors in it makes it stand out more than."	Person is a superfluous thing???
Pt 14 " Direct, right to the point, and the sunlight is like more, it seems to me, because of the colors, it's more bold"	Direct and to the point
Pt 15 " A good reason, it's got warning."	Warning is eye catching. Size is larger, stands out
Pt 16: " Go to warning, and I don't know if they didn't highlight warning or something of that nature, to really drive home the point. I like having the person in that, but, again, my, first is the warning is not on the, I think if the first one had warning on it, I probably would've gone with one, but."	Likes the graphic better in 1. Would prefer to have the person in 2.
Pt 17:	The message and the wordings do not seem to incite a lot of difference . Person is superfluous, ??? yellow is more suitable for sun
No preference	
Pt 2: Both are good	
