# Supplementary material for: Refining Prescription Warning Labels Using Patient Feedback: A Qualitative Study
Source: PLoS One. 2016 Jun 3;11(6):e0156881. doi: 10.1371/journal.pone.0156881 (PMC4892508; doi:10.1371/journal.pone.0156881)
Supplement: S4 Table — (PDF) [file pone.0156881.s004.pdf]

Fig 4: Do not chew or break Label.

Choice and Quotes	Memo
Label 4	
Pt 1”gives an additional warning for me. For the background picture, it kind of draws me to that first.”<br>“I just don't like big pictures on my stuff. I just like this really linear-looking . . .”	The yellow behind the picture is more appealing. Linear looking than others?
Pt 6: “And swallow this medication whole. Do not chew or break. There's some better description there than number one and two and three. And they all look alike. Except these got the warning signs on them too.	Presence of warning sign, clearer words.<br>Yellow is better showing??
Pt 12”Because you can see like more clearly what this picture is telling you about how you can take a pill and like when to take it or so, you know, instead of like during . . . trying to break it or so and trying to share with another person”	Better clarity of picture?, combination of yellow and red/white coloring is alerting. Number 1 is just white. Others are way too bright.
Pt 20:”I think for people that are not, like are lazy readers, just having that background yellow makes it a big difference.”	Yellow attractive. The picture was enough. Prefers the word warning.
Label 1	
Pt 2 Label 1 and 2: “Yeah, because they're big. One, the picture when it shows you how to take the medication. It's in yellow. And in the one, the writing is in yellow. So either or, it works out.”I don't like the small labels. It's hard to really look at the prescription. Like I said, you probably have to spin the bottle around to find those warning labels. I just like to look, soon as you get it, you see your name on the label, on the pill bottle. It should be right there in front of the pill bottle. And that you have to not turn it all the way around just to find the warning label. I like it right there in plain sight.	Size, yellow is any form is fine.<br>Larger label is much better- no need for spinning the bottle. Should be visible to plain sight.
Pt 9”it shows the whole pill and the guy doing it. And the other one, you got to read it and wonder what they're doing.”	Likes the picture of the person swallowing the pill, the other ones require more understanding, Its
Pt 11 “ Well, because then I know this is the do not sign. And I can see that the pill was broken. And I can see the pill goes into your mouth.”	Can see the do not break or chew sign. Circle through line sign is alerting.?? The other labels are not clear
Pt 14 “ It's bold, right to the point”	Prefers yellow behind the text. Highlights more.
Pt 17: “ I guess I'm just particular about that format with the words, the written warning at the bottom of the picture. And then again, you've got the yellow background behind the	Yellow behind the text, the word warning

words that makes the little fine black print stand out.”	
Pt 19:” But the larger head size, even though it’s just a tiny little cartoon, helps me relate to it more because it helps personify the head because it’s larger than the others, so slightly closer to lifelike. “ we’re used to looking at Charlie Brown in the newspaper and things like that. We see the drawings, cartoons of people and have the captions below, you know, the picture, so it’s kind of a familiar scene to see that, I guess.	Size of the head is more “lifelike”. Yellow behind the words<br><br>Cartoons (head of person) feel more relatable
Pt 21: “good because this one, the letters, the instructions are not that big, and so they highlighted with the yellow. That’s perfect.	A big picture doesn’t need yellow highlighting, so the words need highlighting
Label 2	
Pt 8	Yellow caught her attention with the picture being highlighted.
Pt 10 “Because it's bigger, and it tells you not to chew the medicine, swallow it whole, and it's yellow.”	Size, yellow, 3,4 are too long
Pt 13 “To me, the picture, having the picture with the yellow behind it, when the yellow's behind the letters I have a harder time reading the letters. It might stand out better in the white to me, so”	Yellow behind the picture is better, appropriately sized, Yellow behind picture is hard to read
Pt 16:”nytime I see like a color difference, then I'm like something's different, then I got to, I need to look at it, so that's where my eyes are. Now it doesn't have the warning sign on it, which I prefer, personally. “	Caught the color difference. More yellow is better. Prefers not to have warning sign on it.
Pt 18 “ it’s more compact. It’s more out there because it’s this, and the picture is there, and it stands out more.”	The big, yellow color sticks out more
Label 3	
Pt 3 “Well, once again, even though this one is, the words is highlighted, I like this one better the way they got it, because most of the time, the pill bottle, the label is going around the bottle. And once you get this one around the bottle you'll be able to read it better than you would these with the words at the bottom.”	Assumes that the label goes around the bottle and it would be easier to see this label with the highlighted words. Prefers yellow behind the text.
Pt 4: “Yeah, the yellow highlight for the words and the word, warning.”	The word warning is great. Yellow highlight on the words.
Pt 5: “when you have more yellow than white, it looks a little more serious, and the size of it, it	More yellow than white, the word warning

looks more like a warning than just having warning up . . . you didn't have warning up under these two, have a warning there and that was just more yellow than white.”	
Pt 7:”It puts the warning in the yellow.” “f you don't understand the picture, then I suppose you could do the whole label in yellow”<br>“ Once you read the words, it's easy to understand the picture.”<br>“And there's that big red circle with the cross out of the pill broken up.”	If the picture is not understood, the words provide a better understanding and they are highlighted in yellow.<br><br>Words and pictures are to be understood together, they reinforce. Pictures best for visually challenged<br>Number 3 is also appropriately sized
Pt 15: “???	Likes the word warning, more yellow is better.
