# Supplementary material for: Refining Prescription Warning Labels Using Patient Feedback: A Qualitative Study
Source: PLoS One. 2016 Jun 3;11(6):e0156881. doi: 10.1371/journal.pone.0156881 (PMC4892508; doi:10.1371/journal.pone.0156881)
Supplement: S5 Table — (PDF) [file pone.0156881.s005.pdf]

Fig 5: Take with a Full Glass of Water Label.

Choice and Quotes	Memo
Label 3	
Pt 2:"gives you a full detail of a glass of water. A full glass of water or half a glass of water. Most pill bottles just say take with water. This one tells you to take a full glass of water."	Emphasis on the full glass of water
Pt 4: "	Others are too small. appropriately sized<br>Likes the faucet in 3 better than 4.
Pt 5: Interviewer said " So number two is too square and you like number three because it's the longer"	Rectangle shape is better
Pt 7: " I just like that faucet better" I'm more old school. I know what that means.	Old new?? with the faucet, appropriately sized
Unsure??<br>Pt 11: "3 or 4"	Notices that text on white is better than text on yellow. Prefers the faucets in 3 or 4
Pt 14: " It's bold color. It's got a faucet. It has a pill. It has a cup, everything. And the letter isn't writing in black. And they have that little red thing there."	Clarity
Pt 16: " than the J, yeah, upside down J to me. And then, I mean, it does say take with a full glass of water, but, again, if people aren't reading or can't read it and they're using pictures, I would be afraid what if they took alcohol or something different besides water?<br>I mean, maybe milk or, I mean, maybe the medicine can't be used with grapefruit juice, and that's what they have. And so I think clearly labeling it with like a faucet, like we want you to drink a glass of water, because, I mean, if I just take that away, I'd be like, oh, you've got to drink a glass of water with the pill, bu	Clarity
Label-2	
Pt 20: " The faucet, yeah. I mean, I can tell here, but it's just more clear that this is actually a faucet, you know."<br>"The cup is still full, so I don't even have to read what's on the bottom. I already know it's one full cup with my pill."	Faucet is clear, clarity in general, would have liked the word "warning" on it.
Pt 21: " Because the faucet there."	Likes the faucet, but wants to make it bigger and wider.
Label 5	

Pt 1: "I just don't like the faucets. Like to me, this looks like an outside faucet on two and three. And my faucets at home don't look like this either. So I don't care for that. And I know this is a glass of water by just looking at it. The same thing with the one and two, it's just, I don't like the top, bottom thing."	Faucets looks outdoorsy,
Pt 9 : " I'm going by the easiest. There's no faucet or anything. It shows the whole glass of water to take, you know. And that's the one I would know what it meant the quickest."	Quick and easy to understand. Some sort of importance going on?? – "the others she would have to look at everything" but this one- as cut and dry as possible.
Pt 10: " Because it tells you to drink a full glass of water, take the pill. The rest of them got a faucet."	Does not like the addition of a faucet
Pt 13: " I guess it kind of depends. This is kind of, would you say American-centric, because if you were like from France or Mexico, you wouldn't know what either of those symbols were, the faucets. I mean, my kitchen faucet looks like that and my outdoor faucet looks like that, but I know in Germany that they don't, so I don't know."	Faucets are not preferred. This is an American label, so all faucets mean water
Pt 17: " But I think that's simple and straightforward. You've got a glass, a beverage plus the pill. And then take this medication with a full glass of water. Simple. I don't think the faucets and the spouts are really necessary. I mean, it's artistic, but I don't think it's necessary for the label."	Simple No need for faucets
Pt 18: " The rest of them are excellent too, but this one has just a full glass of water for this pill . . ."	Clear that this was a full glass of water. Yellow is alerting
Pt 19: " I don't think that the spigot or faucet needs to have anything to do with it. A glass full of a bluish liquid in a 16-bit color icon is pretty much usually water, so I'm not confused about that being water. "	Clear that the picture of the glass is water. No need for faucet.
Label 1	
Pt 3: "u're going to be able to see the water, the pill plus the writing here" "once you wrap it around the bottle, this is going to be on one side, this is going to be on the other side. I like this one better, because you can see it all right there together."	Faucets are not required. Unnecessary.<br>One glance viewing of all the directions.<br>Bold and attention seeking
Pt 8: " Because it just brought my eye to it again because of the more yellow on it." "	More the yellow, better faucet design

RESPONDENT: And it's over here, where I like that. I think it's all more together right there.”	
Pt 15: “ 5 or 1” “ it looks easier to get it on your bottle without …”	Less to think about in 5. “But these three here, now you would have to wrap them around your bottle, and it would show these three things, and they might not even read it. That's how I look at it.”<br>Wrapping is not a problem
Label -4	
Pt 6: “they don't have the warning sign on them like all the other ones. It's just got the red square or whatever. Where's the warning sign? Warning, warning, warning.”	Presence of the warning sign
Pt 12 “ Because it's like telling you like how they do it and which way to go, because it's more brighter or so with the yellow. Oh, and then they put the white little faucet and a little blue right there . . . and then they put the pill that telling you okay, well, this for me to take with water or like the first one, that's too small.”	Clarity, clear directions, appropriately sized<br><br>Hard to tell if its water in number 2<br><br>White faucet is clearer that its water
